# Seasonal, Sex- and Plant Size-Related Effects on Photoinhibition and Photoprotection in the Dioecious Mediterranean Dwarf Palm, *Chamaerops humilis*

**DOI:** 10.3389/fpls.2016.01116

**Published:** 2016-07-28

**Authors:** Melanie Morales, Marta Pintó-Marijuan, Sergi Munné-Bosch

**Affiliations:** Departament de Biologia Vegetal, Facultat de Biologia, Universitat de BarcelonaBarcelona, Spain

**Keywords:** dioecy, environmental stress, mediterranean, photoprotection, seasonal dynamics, winter photoinhibition

## Abstract

In Mediterranean-type ecosystems plants are exposed to several adverse environmental conditions throughout the year, ranging from drought stress during the warm and dry summers to chilling stress due to the typical drop in temperatures during winters. Here we evaluated the ecophysiological response, in terms of photoinhibition and photoprotection, of the dioecious Mediterranean palm, *Chamaerops humilis* to seasonal variations in environmental conditions. Furthermore, we considered as well the influence of plant size, maturity, and sexual dimorphism. Results showed evidence of winter photoinhibition, with a marked decrease of the *F*_v_*/F*_m_ ratio below 0.7 between January and March, which was coincident with the lowest temperatures. During this period, the de-epoxidation state of the xanthophyll cycle and zeaxanthin levels increased, which might serve as a photoprotection mechanism, owing the full recovery from winter photoinhibition during spring. Furthermore, mature plants showed lower chlorophyll levels and higher β-carotene levels per unit of chlorophyll than juvenile plants, and females displayed lower leaf water contents and higher photoinhibition than males during summer, probably due to increased reproductive effort of females. However, neither low temperatures during winter nor reproductive events in females during the summer led to irreversible damage to the photosynthetic apparatus. We conclude that (i) the Mediterranean dwarf palm, *C. humilis*, suffers from photoinhibition during winter, but this is transient and does not lead to irreversible damage, and (ii) females from this plant species are more sensitive than males to photoinhibition during reproductive events.

## Introduction

Different regions of the world are characterized by the so-called Mediterranean-type ecosystems, with generally warm and dry summers, and wet and mild winters, which determines a great diversity in vegetation that is specifically known as “chaparral” in California, “fynbos” in the Cape Province of South Africa, “matorral” in Chile, “malle” in Australia, and “macchia” (or “maquis”) in the Mediterranean basin (Cody and Mooney, 1978). Plant species distribution in Mediterranean macchias appear to be mainly limited by drought stress, that is a combination of water deficit, high temperatures and high solar radiation during the summer, but it has been suggested that low temperatures in winter may also play a role in plant adaptation and fitness (Mitrakos, 1982). However, most of the ecophysiological research on this macchia vegetation has been focused on the effects of drought stress during summer, and very few studies have investigated thus far the response and adaptation of Mediterranean plants to low-temperature winter stress (Martínez-Ferri et al., 2004; Verhoeven, 2014; Esteban et al., 2015; Míguez et al., 2015).

Although Mediterranean ecosystems are generally considered to be characterized by hot and dry summers, and wet, mild winters, previous studies have already shown sustained decreases in the maximum efficiency of photosystem II (PSII) photochemistry (*F*_v_*/F*_m_ ratio) not only during summer drought, but also during winter, the so-called “winter photoinhibition”, in some evergreen species (Kyparissis et al., 1995; García-Plazaola et al., 1999b; Oliveira and Peñuelas, 2000; Martínez-Ferri et al., 2004; Valladares et al., 2005). Low temperatures during winter may lead to an impairment of the photosynthetic apparatus leading to reductions in PSII efficiency (Berry and Bjorkman, 1980; Adams et al., 1994; Demmig-Adams and Adams, 2000; Yamori et al., 2014), since chilling temperatures strongly reduce photosynthetic activity (Huner et al., 1998; Du et al., 1999; Allen and Ort, 2001; Foyer et al., 2002; D’Ambrosio et al., 2006; Grennan and Ort, 2007; Mohanty et al., 2007), an effect that may be exacerbated on bright days (Öquist and Huner, 1993; Huner et al., 1998). Despite solar radiation decreases considerably during winter (approximately by one-half compared to yearly maxima during late June), Mediterranean plants can still absorb more solar energy that they can use for photosynthesis, particularly when low temperatures are interacting with other stressors, such as drought or abrasive damage due to wind that can strongly enhance foliage cuticular water loss, increasing potential desiccation stress (Grace, 1977; Larcher, 2003). Indeed, drought events are becoming more and more unpredictable and are increasingly occurring during winters in Mediterranean-type ecosystems in the frame of global change (Intergovernmental Panel on Climate Change [IPCC], 2014).

Aside from seasonal variations in environmental conditions that determine plant performance in the Mediterranean macchia, it is also important to consider that some of these plant species show sexual dimorphism. It is generally thought, that females, due to a higher investments in reproductive structures may suffer more photoinhibition and photo-oxidative stress than males under environmental stresses, particularly drought stress (Li et al., 2004, 2007; Rozas et al., 2009; Simancas et al., 2016), but also low temperatures (Xu et al., 2008b; Zhang et al., 2011). However, some exceptions exist, in which no differences between sexes can be observed or even that females outperform males in their tolerance to abiotic stress, so that more studies are required to better understand secondary sexual dimorphism in plants (Barrett and Hough, 2013; Juvany and Munné-Bosch, 2015; Munné-Bosch, 2015).

Mediterranean plants have evolved complex mechanisms of photoprotection to prevent photoinhibition and irreversible damage to the photosynthetic apparatus. When excited states of chlorophyll (^1^Chl^∗^) are not readily processed by photochemistry (photosynthesis), they are converted to triplet excited states (^3^Chl^∗^) and the energy may be transferred to oxygen producing singlet oxygen (^1^O_2_). This reactive oxygen species may eventually cause photo-oxidative stress, thus leading, among other processes, to the inactivation of PSII and/or an increased peroxidation of membrane lipids (Pintó-Marijuan and Munné-Bosch, 2014). To prevent ^3^Chl^∗^ and the subsequent singlet oxygen (^1^O_2_) formation, a carotenoid (xanthophyll cycle)-dependent dissipating pathway is activated to safely return ^3^Chl^∗^ to its ground state. The excess excitation energy is thereby dissipated as heat, directly within the carotenoid protein complexes, bound to the light-collecting chlorophylls (Demmig-Adams and Adams, 2000). Since low temperatures decrease the rate of photosynthesis and increase the excitation energy in chloroplasts; xanthophyll cycle-dependent energy dissipation, which operates in the antenna complexes of PSII, is considered one of the most efficient mechanisms to protect the photosynthetic apparatus (Adams et al., 1994; Ottander et al., 1995; Demmig-Adams and Adams, 2000). Therefore, the role of carotenoids, particularly those of the xanthophyll cycle and zeaxanthin in photoprotection, are considered to be essential in excess energy dissipation and are generally associated with reversible photoinhibition (García-Plazaola et al., 1997, 1999b; Kyparissis et al., 2000).

Singlet oxygen is formed under adverse climatic conditions when the xanthophyll cycle-dependent energy dissipation system is not activated or when it cannot dissipate more excess energy in chloroplasts. ^1^O_2_ can then be eliminated both physically and chemically (the so-called “quenching” and “scavenging” of ^1^O_2_) by the action of antioxidants, which react more quickly than lipids with ^1^O_2_. Carotenoids, and most particularly β-carotene and zeaxanthin, and α-tocopherol (which belongs to the vitamin E group of compounds) eliminate ^1^O_2_. α-Tocopherol, in turn, inhibits the propagation of lipid peroxidation in thylakoid membranes by reacting with lipid peroxyl radicals and therefore preventing the oxidation of poly-unsaturated fatty acids in cascade (Munné-Bosch and Alegre, 2002). Indeed, previous studies have shown that β-carotene and α-tocopherol act synergistically to protect PSII efficiency from ^1^O_2_-induced damage (Trebst, 2003).

The Mediterranean dwarf palm (*Chamaerops humilis* L.) is one of the only two native palms in Europe and the only one in the Iberian Peninsula, where it is autochthonous. Distinctively, this palm is also one of the very few palms originally from a temperate zone and not from the tropics, where most palms are abundant and grow naturally, being able to reach latitudes of up to 44°N (Merlo et al., 1993). This palm is native from continental Europe being mainly found in the western Mediterranean basin, all over the Mediterranean coast of Spain and Portugal, central and southern Italy, some parts of the southern coast of France, islands of the western Mediterranean and northwest Africa (from Morocco to Libya, Global Biodiversity Information Facility [GBIF], 2016). *C. humilis* appears in all phases of the succession of the degradation of Mediterranean ecosystems due to its tolerance to disturbances, such as deforestation, fires, or grazing (Santiesteban et al., 1992; Götzenberger et al., 2003; Alados et al., 2004). Furthermore, it is generally used in gardening, in preference to other palms of alien origin.

Here, we hypothesized that *C. humilis* is very well adapted to the Mediterranean climate and will therefore not suffer photoinhibition and photo-oxidative stress due to the activation of efficient photoprotection mechanisms neither during summer drought nor during winter-associated low-temperature stress. An emphasis was put on the study of photoinhibition and photoprotection mechanisms throughout seasonal variations and the possible influence of plant size, maturity and sexual dimorphism during reproductive events. Our final goal was to improve our knowledge about the ecophysiological response of this unique, native palm from Europe, which is autochthonous in the Iberian Peninsula and is considered a protected species in certain parts of its distribution area (European Food Safety Health [EFSA], 2014).

## Materials and Methods

### Studied Species, Site Description, and Sampling

The native Mediterranean dwarf palm *C. humilis* L. (*Arecaceae*) is a phanerophyte typical of the thermophilic vegetation found in the western Mediterranean basin. From a physiognomic point of view, it is one of the most important determinants of the natural landscape of the coastal macchias. This palm abounds in Mediterranean regions with an accumulated rain above 400 mm and it is more commonly found as part of thickets and spiny shrublands, not just because these are drier areas, but also because of the current deterioration of the Mediterranean vegetation caused by the action of human activities. *C. humilis* is a multi-stemmed shrub with short trunks under natural growth conditions (usually 1.5 m tall maximum), hence its name “dwarf palm.” However, under more optimal growth conditions like gardens, it can reach heights of 4 or even 6 m. The leaves, which emerge in a terminal tuft, have long woody stalks armed with thorns and fan-shaped blades which fold along the midribs (Merlo et al., 1993). The flowering period is in spring, typically from April to May. The flowers appear in dense, short inflorescences at the tops of the stems. The plants are dioecious with male and female flowers on separate plants. The fruit is a globular reddish-brown drupe, oblong or ovoid, measuring 1-4 cm. Unripe fruits are bright green, turning from dull yellow to brown as they ripen during later summer and autumn (September–November).

**FIGURE 1:**
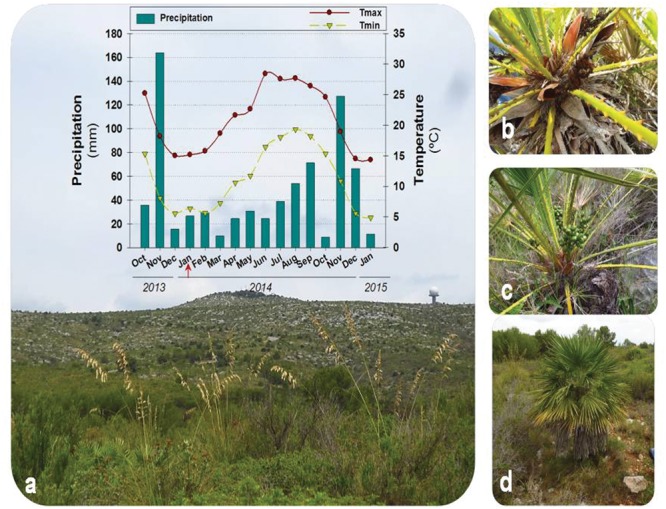
**Seasonal dynamics in environmental conditions during samplings and detail of plants.** Variations in monthly mean maximum and minimum temperatures and precipitation during the period of samplings (January 2014 to January 2015, including 3 months prior to experiments) at the Garraf Natural Park **(a)**, with a detail of a male **(b)**, a female **(c)**, and the tallest plants sampled **(d)**. The red arrow indicates the start of experiments (January 2014).

The present study was carried out on the Garraf Natural Park, one of the 12 natural areas of the Network of Natural Parks of the Barcelona Provincial Council, located near Barcelona (41° 16.443′ N, 1° 55.120′ E; north–east Spain) at 345 m a.s.l. Climatological conditions during samplings were registered in a meteorological station in the same Natural Park at 161 m a.s.l. (41° 15.0′N, 01° 46.0′E). Mean monthly precipitation during the experiments was 40.3 mm (from January 2014 to January 2015, see **Figure 1**), but most rain accumulated during autumn, while the other seasons were quite dry. The hottest month was June, with a mean maximum monthly temperature of 28.4°C, while the coldest month was January, with a mean minimum monthly temperature of 5.0°C (**Figure 1**).

**FIGURE 2:**
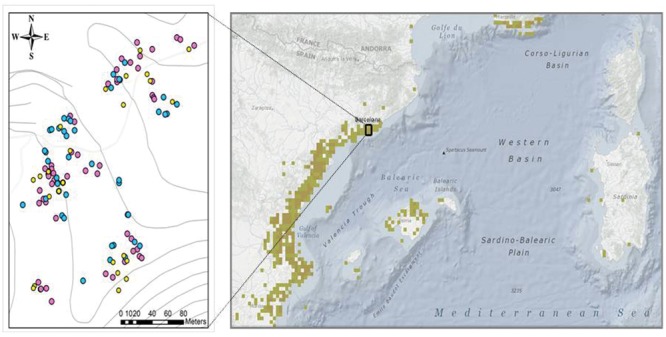
**Absence of spatial segregation in dwarf palms n the Garraf Natural Park.** Distribution of plants sampled from January 2014 to January 2015 at the field site, indicating their exact GPS location **(left)** of juveniles (yellow spots), males (blue spots), and females (pink spots) and location of the population within a detail of macchia Mediterranean distribution **(right)**.

To study seasonal effects on photoinhibition and photo-protection, 12 randomly-selected *C. humilis* individuals were sampled every 2 months at midday (at maximum daily incident solar radiation) on sunny, clear days from January 2014 to January 2015. To study size, maturity and sex-related effects, an additional sampling was performed in 35 juvenile, 35 male, and 35 female randomly-selected plants. Individuals, with a height ranging between 30 cm to 170 cm and georeferenced in the study area (by using Google Earth Pro software, **Figure 2**) were sampled on a sunny, clear day during June 2014. Plant height was measured in every individual to estimate plant size. Fully expanded, mature leaves with no visual damage were collected for measurements, frozen *in situ* in liquid nitrogen and immediately transported to the laboratory, where they were stored at – 80°C and later used for biochemical analyses.

### Leaf Water Content, LMA and *F*v/*F*m Ratio

Leaves were collected, transported to the laboratory in thermal bags at about 4°C in darkness, and weighed to estimate fresh weight (FW). Then, leaves were immersed in distilled water for 24 h at 4°C and weighed for turgid weight (TW). Thereafter, leaves were dried at 80°C until constant weight to determine dry weight (DW). Relative leaf water content (RWC) was calculated as 100 × (FW - DW)/(TW - DW) and, in the same leaves, leaf mass area (LMA) was calculated by measuring its DW and leaf area (g DW/m^2^).

The *F*_v_*/F*_m_ ratio was estimated following Van Kooten and Snel (1990). For this purpose, we used chlorophyll fluorescence data obtained with a portable fluorimeter (Mini-PAM; Walz, Effeltrich, Germany) in leaves maintained for at least 1 h in darkness.

### Photosynthetic Pigments and Photoprotection

The levels of photosynthetic pigments (including chlorophylls and carotenoids) and tocopherols were measured by high performance liquid chromatography (HPLC). In brief, leaf samples were ground in liquid nitrogen and extracted with cold methanol containing 0.01% butylated hydroxyltoluene using ultrasonication. After centrifuging at 12000 rpm for 10 min at 4°C, the supernatant was collected and the pellet re-extracted with the same solvent until it was colorless; then, supernatants were pooled and filtered. Chlorophylls and carotenoids were separated on a binary-solvent gradient using reverse-phase HPLC system and quantified with a diode array detector as described by Munné-Bosch and Alegre (2000). Shortly, pigments were separated on a non-endcapped Zorbax ODS-5 mm column (250 mm long, 4.6 mm i.d., 20% Carbon, Teknokroma, St. Cugat, Spain) at 30°C for 38 min at a flow rate of 1 mL⋅min^-1^ and the injection volume of 80 μL. The solvent mixture for the gradient consisted on (A) acetonitrile:methanol (85:15, v/v) and (B) methanol:ethylacetate (68:32, v/v). The gradient used was: 0–14 min 100% A, 0% B; 14–16 min decreasing to 0% A, 100% B; 16–28 min 0% A, 100% B; 28–30 min increasing to 100% A, 0% B; and 30–38 min 100% A, 0% B. Detection was carried out at 445 nm and compounds were identified and quantified as described previously (Munné-Bosch and Alegre, 2000). On the other hand, tocopherols were separated isocratically on a normal-phase HPLC system and quantified with a fluorescent detector as described by Amaral et al. (2005). The HPLC equipment consisted on an integrated system with a Jasco PU-2089 Plus pump, a Jasco AS-2055 Plus auto-sampler and a FP-1520 fluorescence detector (Jasco, Tokyo, Japan). Tocopherols were separated on an Inertsil 100 A (5 μm, 30 × 250 mm, GL, Sciences Inc, Tokyo, Japan) normal-phase column, operating at room temperature. The flow rate was 0.7 mL⋅min^-1^ and the injection volume was 10 μL. The mobile phase was a mixture of n-hexane and p-dioxane (95.5:4.5, v/v). Detection was carried out at an excitation of 295 nm and emission at 330 nm. Quantification was based on the results obtained from the fluorescence signal and compared to that of a calibration curve made with authentic standard (Sigma–Aldrich, Steinheim, Germany). α-Tocopherol was the only tocopherol homologue present in *C. humilis* leaves.

### Lipid Peroxidation

The extent of lipid peroxidation was estimated from the amount of malondialdehyde acid (MDA) in leaves, following the method described by Hodges et al. (1999), which takes into account the possible influence of interfering compounds in the thiobarbituric acid-reactive substances (TBARS) assay.

### Statistical Analyses

Seasonal variation effects were tested by one-factorial analyses of variance (ANOVA) using Duncan’s *post hoc* tests to identify differences over time. To determine the effect of maturity and sex during reproduction (June), mean values were compared between mature and juvenile plants, and between male and female plants, by using a Student’s *t*-test. Spearman rank’s correlation analyses were performed between plant size (estimated as plant height) and all studied parameters. In all cases, effects were considered significant at a probability level of *P* < 0.05. All statistical tests were performed using the SPSS package (Chicago, IL, USA).

## Results

### Seasonal Dynamics in Photoinhibition

**FIGURE 3:**
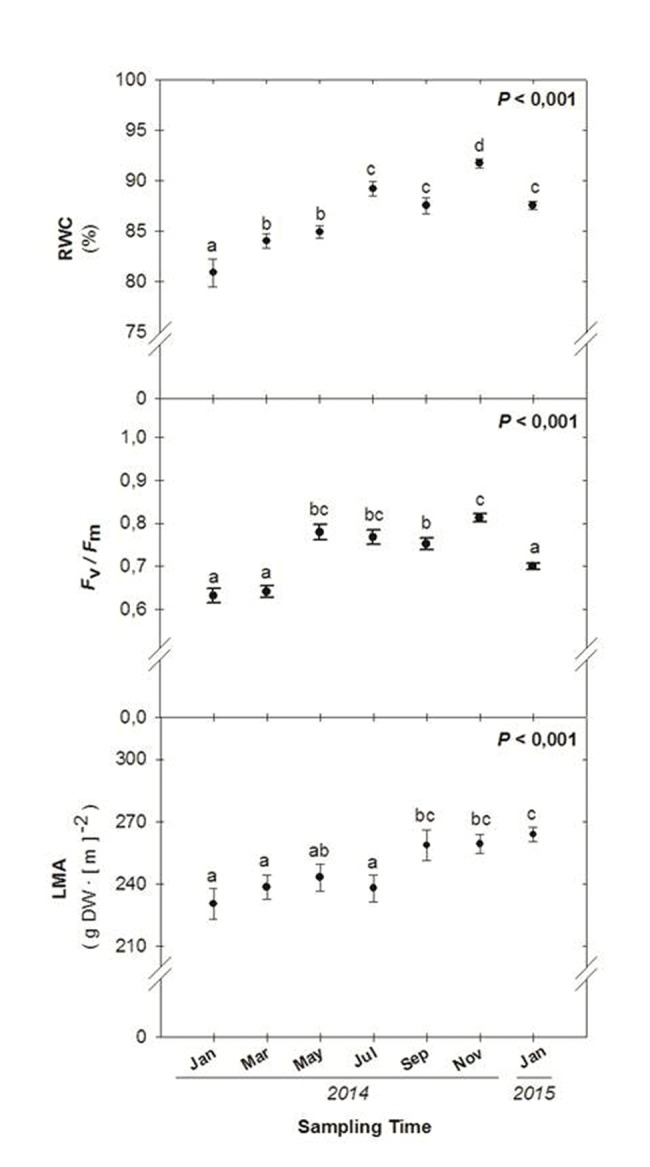
**Seasonal variations in the relative leaf water content (RWC), the maximum efficiency of PSII phochemistry (*F*_v_*/F*_m_ ratio) and leaf mass per area ratio (LMA) in leaves of *Chamaerops humilis*.** Seasonal differences were tested by one-factorial analyses of variance (ANOVA). Different letters indicate significant differences between months using Duncan’s *post hoc* tests. Data represent the mean ± SE of *n* = 12 individuals. Significant *P* values (<0.05) are bold.

Seasonal variations in environmental conditions during the study were typical of the Mediterranean climate with a warm, dry summer and a wet, mild – relatively cold – winter, with most precipitation concentred in autumn, particularly during November (**Figure 1**). Mean monthly maximum temperatures above 25°C and low precipitation occurred during the summer months, although rainfall was progressively increasing from June to September. In contrast, winter months were characterized by mean monthly minimum temperatures below 10°C. The start of experiments (January 2014) coincided with a cold period (with mean monthly minimum temperatures below 7.5°C during the preceding 2 months), and low water availability. Although rainfall accumulated 162 mm during November, December was unexpectedly very dry (17 mm, **Figure 1**). Then, monthly rainfall increased progressively but still was low up to late summer and next November (**Figure 1**). Although the RWC was always kept above 80% throughout the study, it showed a significant increase from 81 to 92% from January to November 2014. The LMA also increased slightly (up to 15%) but significantly throughout the study, with minimum values obtained in January 2014 and maximum values obtained during 2015 (**Figure 3**).

Winter photoinhibition occurred in *C. humilis* leaves. The lowest *F*_v_*/F*_m_ ratios were observed during winter (from January to March 2014, and during January 2015, with values ranging 0.63–0.64 and 0.70, respectively, **Figure 3**). Full recovery of the *F*_v_*/F*_m_ ratio was observed just after the winter 2014, with values ranging between 0.77 and 0.81 during May and November 2014, with mean values around 0.8 during spring, summer, and autumn (**Figure 3**).

In summary, winter photoinhibition (with *F*_v_*/F*_m_ ratios below 0.75) occurred in *C. humilis* leaves (**Figure 3**), but this photoinhibition was transient and occurred in parallel with a drop of temperatures during winter (**Figure 1**), being more evident on drier winters (both the *F*_v_*/F*_m_ ratio and precipitation were lower during winter of 2014 than January 2015, **Figures 1** and **3**).

### Seasonal Dynamics in Photoprotection

**FIGURE 4:**
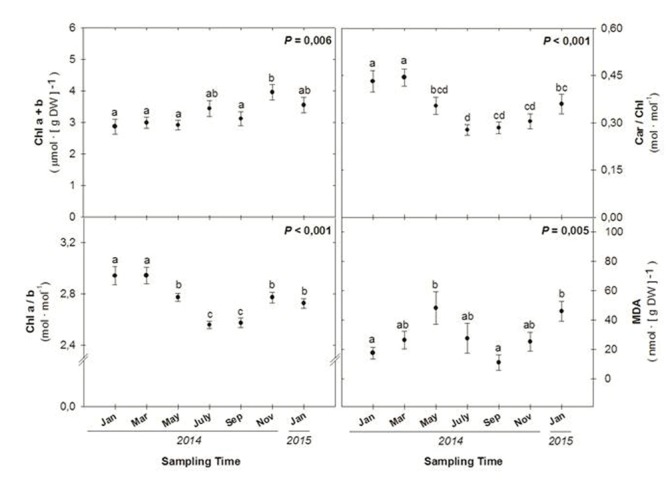
**Seasonal variations in the amounts of photosynthetic pigments (chlorophylls and carotenoids), anthocyanins and malondialdehyde acid (MDA) in leaves of *C. humilis*.** Seasonal differences were tested by one-factorial ANOVA. Different letters indicate significant differences between months using Duncan’s *post hoc* tests. Data represent the mean ± SE of *n* = 12 individuals. Significant *P* values (<0.05) are bold.

While total chlorophyll levels increased throughout the study (**Figure 4**), in parallel with increases in the RWC (**Figure 3**), the Chl a/b decreased during the summer months, reaching minimum values during July and September 2014 (**Figure 4**). The ratio of total carotenoids (Car) per unit of Chl decreased also during the summer in parallel with reductions in the Chl a/b ratio to recover later (**Figure 4**). Malondialdehyde (MDA) levels showed significant seasonal variations, with maximum levels attained during May 2014 and January 2015 (**Figure 4**).

**FIGURE 5:**
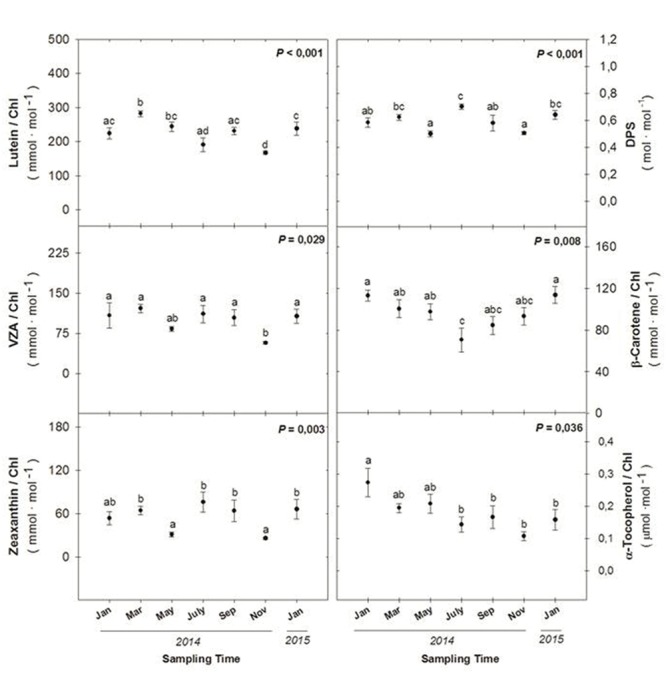
**Seasonal variations in the levels of lutein, xanthophyll cycle pool (VZA), zeaxanthin (Z), de-epoxidation state of the xanthophyll cycle (DPS), β-carotene, and α-tocopherol per unit of chlorophyll in leaves of *C. humilis*.** Seasonal differences were tested by one-factorial ANOVA. Different letters indicate significant differences between months using Duncan’s *post hoc* tests. Data represent the mean ± SE of *n* = 12 individuals. Significant *P* values (<0.05) are bold.

As the Car/Chl ratio increased during winter photoinhibition (**Figure 4**), the levels of specific carotenoids were determined by HPLC, including the levels of xanthophylls (lutein, the xanthophyll cycle pool – VZA – and zeaxanthin), the de-epoxidation state of the xanthophyll cycle (DPS) and β-carotene (**Figure 5**). Maximum levels of VZA, zeaxanthin and the DPS were attained during winter 2014 (January and March), summer 2014 (July and September) and again during winter 2015 (January). In contrast, maximum levels of β-carotene per unit of chlorophyll were attained during the winters 2014 and 2015 (January in both cases), and maximum levels of α-tocopherol per chlorophyll unit were attained during January 2014, decreasing progressively throughout the experiment (**Figure 5**), inversely with the RWC (**Figure 3**).

In summary, the DPS increased both during winter and summer, while β-carotene per unit of chlorophyll increased during winter, and α-tocopherol per chlorophyll unit inversely correlated with the RWC.

### Influence of Maturity, Dioecy, and Size

The influence of maturity, sex, and size on all measured parameters was specifically evaluated during the reproductive stage, particularly during June 2014. Mature plants showed higher LMA, lower chlorophyll levels, and higher chlorophyll a/b ratio and β-carotene per unit of chlorophyll than juvenile plants (**Table 1**). These differences were observed both in males and females, since these parameters (LMA, chlorophylls and β-carotene) did not differ between sexes. In contrast, sexual dimorphism was evidenced on the leaf water contents and the *F*_v_/*F*_m_ ratio, females showing slightly lower leaf water contents and higher photoinhibition than males during the summer (**Table 1**).

**Table 1:** Influence of maturity and sex on all measured parameters in the Mediterranean palm, *Chamaerops humilis*, under field conditions during their reproductive stage (June 2014).

	Maturity		Sex	
	Juvenile	Mature	Male	Female
RWC (%)	82.4±0.66	84.1±0.73	85.70±0.96	82.60±1.00 ˆ*
LMA (g⋅DW⋅m^-2^)	242.86±5.94	276.76±3.11ˆ*	279.71±4.77	273.75±4.00
*F*_v_*/F*_m_	0.65±0.02	0.64±0.01	0.67±0.02	0.67±0.02*
Chl a+b (μmol⋅g DW^-1^)	2.96±0.15	2.52±0.08ˆ*	2.52±0.10	2.51±0.12
Chl a/b	2.64±0.03	2.78±0.02ˆ*	2.78±0.03	2.76±0.02
Car/Chl (mol⋅mol^-1^)	0.35±0.02	0.35±0.01	0.34±0.02	0.36±0.02
MDA (nmol⋅g DW^-1^)	52.90±9.30	50.03±3.98	57.28±6.64	43.00±3.98
Lut/Chl (mmol⋅mol^-1^)	257.7±11.6	255.3±7.54	262.71±83	248.02±1.02
VZA/Chl (mmol⋅mol^-1^)	92.28±6.94	90.93±5.15	88.47±8.38	93.31±6.17
Z/Chl (mmol⋅mol^-1^)	58.91±6.33	55.73±4.68	51.62±7.39	59.71±5.83
DPS (mol⋅mol^-1^)	0.60±0.03	0.56±0.03	0.52±0.04	0.61±0.03
β-Car/Chl (mmol⋅mol^-1^)	95.34±9.86	112.05±3.63ˆ*	113.35±3.72	110.79±4.59
α-Toc/Chl (μmol⋅mol^-1^)	0.47±0.05	0.41±0.03	0.39±0.04	0.42±0.04

*Data correspond to the mean ± SE *n* = 35 juvenile and *n* = 70 mature plants, *n* = 35 male and *n* = 35 female plants. An asterisk indicates differences between mature and juvenile plants; male and female plants (Student’s*t*-test, *P* < 0.05). RWC, relative water content; LMA, leaf mass per area ratio; *F*_v_*/F*_m_, maximum efficiency of photosystem II photochemistry; Chl, chlorophyll; Car, carotenoids; MDA, malondialdehyde acid; Lut, lutein; VZA, xanthophyll cycle pool; Z, zeaxanthin; DPS, de-epoxidation state of the xanthophyll cycle; β-Car, β –carotene; α-Toc α-tocopherol.*

Plant size (estimated as plant height) positively correlated with the LMA, chlorophyll a/b and carotenoid/chlorophyll ratios, particularly in the case of lutein and β-carotene, and negatively correlated with total chlorophyll levels (**Table 2**). The strongest correlation was observed for the LMA (*r* = 0.371, *P* < 0.001). In all other cases, correlations were weaker, with r values below 0.3 in all cases (**Table 2**). Indeed, if Bonferroni correction was applied to the data, the *P* value would decrease to 0.003, correlations being therefore significant for the LMA and total chlorophyll levels only. In other words, the largest plants investigated (with a height of up to 1.7 m) tended to have thicker leaves (as indicated by the LMA) and reduced chlorophyll contents compared to smaller individuals.

**Table 2:** Coefficient (*r*) and *P*-values (shown in parentheses) of Spearman’s rank correlation between the plant size (height) and all parameters analyzed during the reproductive stage (June 2014, *n* = 105).

RWC	0.226 (0.010)
LMA	**0.371 (<0.001)**
*F*_v_*/F*_m_	**-**0.066 (0.252)
Chl a+b	**-0.278 (0.002)**
Chl a/b	**0.277 (0.011)**
Car/Chl	**0.174 (0.040)**
MDA	**-**0.064 (0.261)
Lut/Chl	**0.232 (0.010)**
VZA/Chl	**-**0.115 (0.125)
Z/Chl	**-**0.097 (0.168)
DPS	**-**0.081 (0.210)
β-Car/Chl	**0.227 (0.011)**
α-Toc/Chl	**-**0.046 (0.322)

*The *P* values below 0.05 are bold. Abbreviations used as in **Table 1***

In summary, females from this plant species are more sensitive than males to photoinhibition during reproductive events. Furthermore, both increased plant size and maturity led to structural changes in leaves (as indicated by increases in the LMA) and changes in the accumulation of photosynthetic pigments (as indicated by the reductions in chlorophyll levels).

## Discussion

In their post-embryonary stage, plants are sessile and cannot escape from adverse environmental conditions, so they have evolved a number of complex, interconnected mechanisms of adaptation to allow their survival. Plants living in Mediterranean-type ecosystems are subjected to marked seasonal variations throughout the year and, consequently, exposed to multiple stresses. Here, it is shown that *C. humilis* can withstand both summer droughts and low temperatures during winter, despite a transient photoinhibition was observed during the winter months. Previous studies have shown that there are two mechanisms to counteract the potential damaging effects of excess solar radiation: (i) prevent or avoid photoinhibition, which is found in species that are able to maintain a sustained PSII photochemical efficiency, and (ii) tolerate photoinhibition, which is observed in species displaying dynamic photoinhibition, which is in turn associated with fast reversible mechanisms (Demmig-Adams and Adams, 1992; Long et al., 1994). The latter include activation of the synthesis of non-epoxy xanthophylls (typically zeaxanthin, although some species additionally accumulate lutein from lutein epoxide), leading to the development of non-photochemical quenching (Martínez-Ferri et al., 2000, 2004; Jahns and Holzwarth, 2012).

Reversible, transient photoinhibition during the winter was manifested by *F*_v_*/F*_m_ ratios below 0.75 during the cold temperatures of January–March in the present study, which was followed by a full recovery to optimal values (*F*_v_*/F*_m_ ratios of 0.8) during spring. *C. humilis* is described here, to our knowledge for the first time, as a photoinhibition-tolerant palm. Furthermore, we show that this tolerance is achieved through the activation of xanthophyll cycle de-epoxidation, leading to an enhanced accumulation of zeaxanthin, as it occurs in other evergreen species from Mediterranean-type ecosystems (García-Plazaola et al., 1999a,b, 2003) and other habitats (Baker, 1995; Verhoeven et al., 1996). In these species, low predawn *F*_v_*/F*_m_ values during winter have been associated with the overnight retention of high amounts of zeaxanthin, which has been attributed to the inhibitory effect of chilling temperatures on the enzymatic conversion of zeaxanthin from violaxanthin in the xanthophyll cycle (Adams and Demmig-Adams, 1994; Adams et al., 1994; Leipner et al., 2000). Thereby, the winter photoinhibition observed in *C. humilis* in the present study can be interpreted as an adaptive photoprotection mechanism of the photosynthetic apparatus, as it occurs in some evergreen conifers, which are adapted to extreme environments, showing the greatest degree of photoinhibition during the winter months (Adams et al., 2004; Demmig-Adams and Adams, 2006).

In an attempt to get some insights into the possible causes and consequences of winter photoinhibition, we correlated the *F*_v_*/F*_m_ ratio with all other measured parameters (**Table 3**). It turned out that the *F*_v_*/F*_m_ ratio not only negatively correlated with the DPS, VZA and zeaxanthin levels, but also with the levels of lutein and β-carotene per chlorophyll unit, and it positively correlated with the leaf water content (see “Seasonal” column, **Table 3**). This indicates that water deficit had some influence on the development of the transient photoinhibition during winter months, which has important biological implications in the framework of global change, in which drought events are becoming more unpredictable. Furthermore, it is interesting to note that this transient photoinhibition was achieved with parallel decreases in the content of chlorophylls, particularly that of chlorophyll b (as indicated by the negative correlation with the chlorophyll a/b ratio, **Table 3**), but increases in the levels of lutein and β-carotene per unit of chlorophyll. Both lutein and β-carotene protect the photosynthetic apparatus from ^1^O_2_-induced damage (Telfer, 2005; Dall’Osto et al., 2006; Triantaphylidès and Havaux, 2009; Ramel et al., 2012, 2013), thus suggesting photoprotection to PSII is achieved by decreasing the amount of photons that enter the photosynthetic electron transport (by reducing chlorophyll levels, particularly those found in the antenna), and by increasing the amount of photoprotective molecules that protect from ^1^O_2_-induced damage. It appears, therefore, that xanthophyll cycle-dependent energy dissipation operates in parallel with mechanisms that increase the elimination of ^1^O_2_. Prevention and elimination operate therefore simultaneously to avoid chronic photoinhibition of the photosynthetic apparatus in *C. humilis*.

**Table 3:** Coefficient (r) and *P*-values (shown in parentheses) of Spearman’s rank correlation between the maximum efficiency of PS II photochemistry (*F*_v_*/F*_m_ ratio) and all other measured parameters.

	All data	Seasonal	Sex
RWC	**0.281 (<0.001)**	**0.397 (<0.001)**	0.025 (0.400)
LMA	**-**0.033 (0.326)	0.041 (0.345)	**-**0.014 (0.444)
Chl a+b	**0.319 (<0.001)**	**0.301 (0.003)**	**0.207 (0.018)**
Chl a/b	**-0.127 (0.042)**	**-0.374 (<0.001)**	0.087 (0.191)
Car/Chl	**-0.352 (<0.001)**	**-0.489 (<0.001)**	**-0.258 (0.004)**
MDA	**-**0.077 (0.146)	0.003 (0.488)	0.037 (0.354)
Lut/Chl	**-0.330 (<0.001)**	**-0.450 (<0.001)**	**-**0.117 (0.122)
VZA/Chl	**-0.375 (<0.001)**	**-0.479 (<0.001)**	**-0.305 (0.001)**
Z/Chl	**-0.304 (<0.001)**	**-0.458 (<0.001)**	**-0.316 (0.001)**
DPS	**-0.339 (<0.001)**	**-0.352 (0.001)**	**-0.319 (0.001)**
β-Car/Chl	**-0.324 (<0.001)**	**-0.401 (<0.001)**	**-0.191 (0.028)**
α-Toc/Chl	**0.144 (0.024)**	-0.148 (0.090)	-0.143 (0.074)

*Correlations have been performed for all pooled data (*n* = 189), and separately for seasonal variations data (*n* = 84) and the reproductive stage (June 2014, *n* = 105). The *P*-values below 0.05 are bold. Abbreviations used as in **Table 1***

The dwarf palm also showed strategies for overcoming severe drought conditions, such as keeping leaf water content always above 80% and the activation of xanthophyll cycle-dependent excess energy dissipation during the summer. It has already been shown that this palm is well adapted to the Mediterranean summer conditions. In a comparative study between dominant species of a coastal Mediterranean macchia ecosystem, the dwarf palm showed greater water potential values, thus indicating a better tolerance to water deficit (Rotondi et al., 2003). As July might be a critical period for this species due to high temperatures, high solar radiation and drought, it is the month with maximum activation of xanthophyll cycle-dependent excess energy dissipation, which acts as a photoprotection mechanism helping to tolerate summer drought as in other typically Mediterranean species (Míguez et al., 2015). These results emphasize the importance of understanding adaptive mechanisms to drought tolerance in these habitats (Bermúdez and Retuerto, 2014; Catoni and Gratani, 2014; Gratani et al., 2014; Lloret et al., 2016). In other palms, it has also been shown enhanced drought tolerance related to greater efficiency in preventing oxidative damage by activating antioxidant mechanisms (e.g., see Gomes and Prado, 2007 for the coconut palm *Cocus nucifera*; Al-Khayri and Ibraheem, 2014 and Arab et al., 2016 for the date palm *Phoenix datilifera;* Silva et al., 2016 for the oil palms *Elaeis guineensis* and *Elaeis oleifera*).

Sexual dimorphism can lead to profound differences in secondary sexual characters, such as vegetative growth and plant response to both biotic and abiotic stresses; but the extent to which these stresses can affect photoinhibition, photoprotection, and photo-oxidative stress in dimorphic plants is still very poorly understood. Previous studies have shown that females are generally more sensitive to photoinhibition that males when subjected to either drought stress (Li et al., 2004, 2007; Xu et al., 2008a,b; Rozas et al., 2009; Chen et al., 2010, 2014; Zhang et al., 2010, 2014; Kumar et al., 2016) or low temperatures (Li et al., 2007; Zhang et al., 2011; Juvany et al., 2014), although some exceptions exist (e.g., see Oñate et al., 2012; Morales et al., 2013; He et al., 2016). In the present study, it was found that females are more sensitive than males to photoinhibition only during the summer. It is interesting to note that sex-related differences disappeared during winter, when plants were not reproducing, thus indicating photoinhibition in *C. humilis* is a clear secondary sexual character associated to an increased reproductive effort in females. This may be associated with sex-related differences in trade-offs in life-history traits, so that plants may allocate resources on growth, reproduction or maintenance (metabolism or defense) (Yang et al., 2014). Since females allocate a greater investment in reproduction, generally showing distinct nutrient requirements (Zhao et al., 2012; Zhang et al., 2014; Chen et al., 2015; Simancas et al., 2016), they decrease resources for other functions like defenses, and this will be the reason why females are more sensitive to photoinhibition that males when they are subjected to drought stress. Despite this, females are able to recover from photoinhibition and sex differences disappear after the reproductive period. This is particularly interesting since it suggests that females simply respond differently than males, showing compensatory mechanisms to overcome enhanced nutrient requirements during reproductive events (Obeso, 2002; Barrett and Hough, 2013; Juvany and Munné-Bosch, 2015). Previous studies have shown sex-related differences in flowering response to photoperiod (Zhao et al., 2009) and source-to-sink transitions (Zhao et al., 2012), which may indeed reflect complex sex-related differences in the physiology of dioecious plants (Ashman, 2006). If environmental stress is mild and/or transient, such compensatory mehanisms may be effective to overcome higher nutrient requirements during reproductive events in females than in males, but when stress is too severe or persists in time, more damage occurs in females than males. Our results suggest that transient photoinhibition during reproductive events in summer in females of *C. humilis* is indeed an adaptive mechanism to prevent irreversible damage. In this respect, it is also noteworthy that *C. humilis* did not show spatial segregation between sexes, as shown in **Figure 2** Previous studies indicate lower ability for competition in females than males, which is associated with increased nutrient requirements in the former (Chen et al., 2014, 2015). In *C. humilis*, at least in our field site in the Garraf Natural Park, sex competition may occur and play a role determining sexual differences in physiological traits, but competition with other plant species, particularly some invasive ones, such as *Cortaderia selloana*, may be indeed even more important. This aspect requires, however, further investigations.

## Conclusion

The present study demonstrates the photoprotective capacity of the unique native Mediterranean palm during winter photoinhibition, in which plants are able to overcome the harmful effects of chilling stress combined with high light. Furthermore, it is shown here that females are more sensitive than males to photoinhibition during reproductive events, although these effects are transient and reversible. Due to the ecological importance of the Mediterranean dwarf palm, the high capacity of photoprotection described here supports the use of this species in reforestation programs, both after fires and to recover degraded soils.

## Author Contributions

MM, MP-M, and SM-B designed the experiments; MM and MP-M performed experiments; SM-B contributed materials and reagents; MM and SM-B wrote the manuscript.

## Conflict of Interest Statement

The authors declare that the research was conducted in the absence of any commercial or financial relationships that could be construed as a potential conflict of interest.
